# Antibiotics and Infectious Respiratory Diseases

**DOI:** 10.3390/antibiotics11070859

**Published:** 2022-06-27

**Authors:** Francesco Di Gennaro, Gina Gualano, Fabrizio Palmieri

**Affiliations:** 1 Respiratory Infectious Diseases Unit, National Institute for Infectious Diseases “L. Spallanzani” IRCCS, 00149 Rome, Italy; gina.gualano@inmi.it (G.G.); fabrizio.palmieri@inmi.it (F.P.); 2 Clinic of Infectious Diseases, Department of Biomedical Sciences and Human Oncology, University of Bari “Aldo Moro”, 70123 Bari, Italy

Respiratory infectious diseases (rIDs) remain among the most significant causes of morbidity and mortality worldwide, and, in the era of COVID-19, they have come into major focus in the scientific world and global health approaches. They can have a viral, bacterial, parasitic or opportunistic origin; however, indiscriminate use of antibiotics in their treatment has led to the development of antimicrobial resistance. Effective management of respiratory infectious diseases to reduce their burden and to avoid overuse of antibiotics has become a great therapeutic and public health challenge. This Special Issue of *‘Antibiotics’* focuses on various aspects of medical and clinical use of antimicrobials in infectious respiratory diseases in real-life settings. Nine articles (including a review article) are included, each focusing on a different issue in an essential field of applied research, furthering the scientific knowledge on this important topic.

Kurotschka and colleagues [1], in an extensive study, underline the role of medical education in appropriate general use of antimicrobials and pediatric primary care in Germany, where the majority of participating primary care physicians (PCPs) have attended at least one specific training course on antibiotic use yet declare they need more training on this topic. Medical education is crucial to contain the spread of antimicrobial resistance. To improve the understanding of the decision-making strategies of general practitioners (GPs), Martínez-González and colleagues [2] performed a very interesting and well-conducted study on the role of C-reactive protein (CRP) point-of-care testing (POCT) in decision making for antibiotic prescription for respiratory infectious diseases. They outline how guidelines and policies helping to improve treatment decisions may benefit from integrating CRP-guided antibiotic prescription recommendations. Similarly, these results could be valuable in countries where CRP-POCT is widely used.

Numerous researchers have focused on the issue of tuberculosis, which remains one of the most common infectious respiratory diseases, especially in low-income countries, with the worst impact on vulnerable populations.

D’ambrosio, with an international team [3], suggests a surveillance system with systematic screening of immigrants from high-TB-burden settings is crucial in TB control, even in countries with a low TB incidence. While their study, involving 80 countries, highlighted this aspect and strategy in TB control, Patti and colleagues [4] studied in-depth the role and impact of vitamin A, B, C, D and E implementation on Tb outcomes. The authors studied immunological, cytological and clinical aspects and demonstrate a key role of the vitamin approach during TB treatment. Their results, which are highly relevant, can help the academic and clinical communities to evaluate the use of vitamins in patients with TB with underlying malnutrition, demonstrating good results not only in TB outcomes but also in sputum conversion.

In addition, Igwaran performed a bibliometric study, underlining how researchers around the world are engaged in the fight against tuberculosis; South Africa was the most studied and cited country for tuberculosis publications [5]. An interesting experience was shared by Di Bari and colleagues [6]. They evaluated 1800 cases of TB from 2016 to December 2021, with 29 patients (1.61%) exhibiting pulmonary thromboembolism (PTE) at admission or during their hospital stay. These researchers found an increased prevalence of PTE between the pre- and post-COVID-19 pandemic periods and reinforce how acute respiratory failure and extensive TB disease increased significantly during the pandemic. They recommend screening for PTE in all TB patients due to this potentially life-threatening complication of TB.

For patients with SARS-CoV-2 pneumonia, Poliseno and the other researchers on the Foggia team present a very relevant case history on the use of remdesivir and its efficacy and safety profile [7]. Their data show a low rate of ICU admission, a high rate of clinical recovery and good drug safety observed in COVID-19 patients treated with remdesivir during two distinct pandemic waves.

The last two articles in our Special Issue focus on two topics of antimicrobial resistance. De Palma shares 20 years of experience in treating mediastinitis, a pathology with high morbidity, mortality and polymicrobial etiology [8].

Meanwhile, Rando and colleagues describe a case series on Acinetobacter MDR infections and the use and efficacy of cefiderocol [9]. Their work is highly relevant and may contribute to redefining cefiderocol’s role in treating severe carbapenem-resistant A. baumannii pneumonia, adding new information from clinical experience with this drug to the current body of knowledge.

We believe this Special Issue will be a source of significant value and inspiration for the clinical and academic communities around the world.
